# Co-developing Georgia’s National Digital Health Strategy through a multistakeholder convergence workshop: An implementation case study from the WHO European Region

**DOI:** 10.1371/journal.pdig.0001787

**Published:** 2026-10-14

**Authors:** Nato Gzirishili, Mehar Grover, Resham Sethi, Mariam Beridze, Tomas Roubal, Lela Sulaberidze, Karapet Davtyan, Silviu Domente, Karthik Adapa

**Affiliations:** 1 World Health Organization Country Office in Georgia, Tbilisi, Georgia, United States of America; 2 Strategic Development and Analytics Department, Ministry of Internally Displaced Persons from the Occupied Territories, Labour, Health and Social Affairs (MoIDPLHSA), Tbilisi, Georgia, United States of America; 3 World Health Organization Regional Office for Europe, Copenhagen, Denmark; 4 World Health Organization Regional Office for South-East Asia, New Delhi, India; 5 University of North Carolina-Chapel Hill, Chapel Hill, North Carolina, United States of America; Neu-Ulm University of Applied Sciences: Hochschule fur angewandte Wissenschaften Neu-Ulm, GERMANY

## Abstract

National digital health strategies (DHS) are widely recognized as essential for advancing universal health coverage. However, published evidence on operational, multi-stakeholder approaches to co-development—particularly beyond the Asian and African settings where convergence methodology has previously been applied—remains limited. This study details the application of a convergence-based methodology to validate and refine the draft National DHS of Georgia, an upper-middle-income country, aligning it with European Union frameworks. An implementation case study was conducted in Georgia from June to September 2025. Central to this was the Multistakeholder Convergence Workshop, adapted from the Asian Development Bank Digital Health Convergence Meeting Toolkit and a four-phase blueprint methodology. Forty-seven participants from diverse sectors (public, private, academia, and development partners) collaborated in five thematic working groups. They reviewed the draft DHS, generating recommendations that were synthesized against the seven WHO-ITU eHealth strategy building blocks using hybrid deductive-inductive thematic analysis by two independent coders with third-party arbitration. The revised strategy comprises six strategic goals and 38 priority activities. Key technical recommendations included adopting a federated data governance model, utilizing the existing national identification number instead of a separate Health ID, and establishing a national Health Information Exchange. The strategy endorses FHIR for data exchange, LOINC for laboratory data, and the WHO-FIC (including ICD-11) complemented by SNOMED CT. Governance recommendations emphasized enacting a stand-alone Digital Health Act, forming a dedicated Digital Health Unit within the MoIDPLHSA, integrating digital health into professional education, and extending the strategy horizon to 2030 with a mid-2026 review checkpoint. Structured convergence methodologies, initially developed for Asian settings, can be effectively adapted for upper-middle-income WHO European Region countries pursuing European Health Data Space alignment. This approach produced technically specific outputs, reinforced national ownership, and supported Georgia’s transition toward implementation, offering a highly transferable framework for similar contexts.

## Author summary

We describe how Georgia developed its first National Digital Health Strategy through a structured multistakeholder convergence workshop—a method previously used to support digital health blueprint development in countries across Asia and Africa. Like many health systems undergoing transition from centralized public models toward mixed public–private delivery, Georgia faces fragmented digital systems, weak interoperability between predominantly private healthcare providers and public platforms, gaps in the digital health workforce, and incomplete legal frameworks. We brought together 47 participants from government, private healthcare providers, academia, professional associations, and development partners into five thematic working groups over two days in July 2025. Each group reviewed a section of the draft strategy and proposed concrete revisions. Together, participants validated the strategy’s vision, mission, and guiding principles, and recommended a federated data governance model, adoption of international interoperability standards, a stand-alone Digital Health Act, a dedicated Digital Health Unit within the Ministry, and stronger pre-service and in-service training. They also extended the implementation horizon from two years to 2030. Our experience suggests that the convergence methodology is portable beyond its original setting and offers a potentially transferable approach for other countries developing or refining national digital health strategies, while implementation effectiveness remains to be evaluated.

## 1. Introduction

Digital health is increasingly recognized as a catalytic enabler of health system strengthening, supporting equitable access to services, reducing healthcare costs, and accelerating progress toward universal health coverage (UHC) [1–4]. The 2005 World Health Assembly resolution WHA58.28 on eHealth, and subsequent resolutions WHA66.24 (2013) on standardization and interoperability and WHA71.7 (2018) on digital health, urged Member States to integrate digital tools into health systems [1,2]. In 2020, the World Health Assembly endorsed the Global Strategy on Digital Health 2020–2025—subsequently extended to 2027—as a unifying framework for health systems strengthening through digital innovation [5,6]. At the regional level, the WHO European Region adopted the Regional Digital Health Action Plan 2023–2030, providing a more operational framework tailored to regional priorities, with a strong emphasis on governance, interoperability, data use, and capacity-building for digital transformation [7]. To date, more than 120 Member States have developed national eHealth or digital health strategies [5]. While the global strategy provides high-level direction, it explicitly emphasizes contextual adaptation, and the regional action plan reinforces country-specific implementation pathways aligned with common regional standards [5,7]; national strategies must therefore reflect local realities while leveraging international standards. Importantly, the global framework was itself developed through wide-ranging consultation, setting a precedent for participatory approaches in shaping national strategies.

Georgia, an upper-middle-income country in the South Caucasus and a candidate for European Union (EU) accession, illustrates both achievements and challenges typical of health systems undergoing structural transition from centralized, state-run models toward mixed public–private financing and delivery arrangements. Public health spending nearly tripled to GEL 2.0 billion between 2014 and 2022, and core public health programs have been progressively digitalized [8]. However, Georgia’s digital health ecosystem remains fragmented [8,9]. Approximately 86% of secondary and tertiary hospital beds are in privately owned facilities, each operating its own hospital management information system with inconsistent standards [8]. At the primary healthcare (PHC) level, only an estimated 20% of facilities use electronic records, and more than 180 medical forms are submitted across multiple, siloed reporting systems [8,9]. The Information Technology Agency (ITA) of MoIDPLHSA maintains more than 70 electronic modules for different government agencies while attempting to consolidate patient records from over 250 hospitals through point-to-point integration; according to World Bank and Asian Development Bank (ADB) assessments, the resulting national electronic health record (EHR) is reported to be frequently incomplete, of variable quality, and seldom used by clinicians in routine care [8,9]. Compounding these challenges are workforce shortages in digital health, the absence of structured pre-service and in-service training pathways, reliance on donor financing, and disparities in digital access between urban and rural settings [9,10]. These challenges closely align with priority areas identified in the Regional Digital Health Action Plan for the WHO European Region 2023–2030, particularly regarding interoperability, governance, workforce capacity, and equitable access [7].

Within this context, Georgia is also pursuing alignment with the European Health Data Space (EHDS) Regulation (Regulation (EU) 2025/327), which entered into force in March 2025 and envisages interoperable, cross-border health data exchange across EU Member States [11]. Achieving such alignment requires a coherent national digital health strategy underpinned by clear governance, modern interoperability standards, and a robust legal and policy framework.

Against this backdrop, the Ministry of Internally Displaced Persons from the Occupied Territories, Labour, Health and Social Affairs (MoIDPLHSA), with technical support from the World Health Organization (WHO) Country Office in Georgia, the WHO Regional Office for Europe, and the WHO Regional Office for South-East Asia (WHO–SEARO), initiated the development of Georgia’s first National Digital Health Strategy (DHS); prior to this process, Georgia had no dedicated national digital health strategy, as documented in the World Bank’s 2022 assessment [8]. The strategy was developed through a multiphase and consultative process that included global benchmarking against nine comparator countries—Australia, Denmark, Estonia, Germany, India, Singapore, the United Kingdom, and regional peers Armenia and Türkiye [12–19]—followed by national contextualization, expert interviews, field visits, and institutional reviews across MoIDPLHSA departments (all listed ministries, departments, and agencies in this paper are Georgian institutions unless otherwise indicated). The process culminated in a Multistakeholder Convergence Workshop convened on 18–19 July 2025.

While the conceptual case for participatory, multi-stakeholder digital health strategy development is well established, practical guidance on operationalization remains limited. The ADB Digital Health Convergence Meeting Tool Kit (2018) [20] provides a widely referenced six-stage framework. It has been applied in several Asian and African countries, including Bhutan, Indonesia, Myanmar, Nepal, Timor-Leste, and Viet Nam [20,21], Sri Lanka [22], and Kenya [23]. Recent work has further described the application of a multi-phase, participatory blueprint development methodology in Maldives, Nepal, and Timor-Leste [24]. However, published evidence on adapting and applying this methodology in WHO European Region settings—particularly in upper-middle-income, EU-aligning countries—is scarce.

This paper addresses that evidence gap. The objectives of this paper are to: (i) document the application of a convergence-based methodology to develop and refine Georgia’s first National Digital Health Strategy; (ii) thematically synthesize workshop outputs against the WHO–ITU eHealth Strategy seven building blocks; (iii) compare Georgia’s experience with prior applications of the convergence methodology in other settings; and (iv) distil lessons for potential transfer to similar upper-middle-income country contexts.

## 2. Materials and methods

### 2.1. Ethics statement

This work constitutes programmatic implementation support provided by WHO to the Georgian health sector and did not involve primary research with human participants, collection of identifiable personal data, or clinical intervention. Consistent with WHO’s policy framework distinguishing research involving human subjects from programmatic and public-health practice activities, the WHO offices involved determined that this activity constituted programmatic implementation support rather than human-subjects research, and formal research ethics committee review was accordingly not sought. Participants were informed at the workshop that its outputs would be documented in aggregate, non-attributable form in programme reports and potential publications. All workshop participants engaged in their institutional capacity. Aggregated, non-attributable workshop-level findings are reported.

### 2.2. Study design

This paper is presented as a retrospective, single-case implementation case study. The unit of analysis is the Multistakeholder Convergence Workshop, examined within the broader national strategy-development process in which it was embedded: the workshop is the primary object of study, and the surrounding process provides its context. We classify the study as an implementation case study—rather than a strategy-development or process case study—because the object of inquiry is the application of an implementation strategy (the convergence methodology) to a defined task, not the content of the strategy document itself. The temporal boundaries of the case, June to September 2025, were set to span the pre-workshop circulation of the draft DHS to participants (15 June 2025), the delivery of the workshop (18–19 July 2025), the integration of workshop feedback, and the follow-up Digital Health Matchmaking and Investment Workshop (September 2025) that concluded the immediate post-workshop phase. The case study draws on workshop documentation generated as part of the programme—structured rapporteur notes, group presentations, and written feedback—together with post-workshop synthesis conducted by the workshop coordination team; this material was generated as programme documentation rather than under a prospective research protocol, and the analysis described in Section 2.5 was applied retrospectively.

### 2.3. Conceptual framework

The strategy development process was anchored in two complementary frameworks that informed both the methodological approach and workshop design. First, the WHO–ITU National eHealth Strategy Toolkit provided the overarching structural foundation for the development of the strategy, organizing digital health across seven interdependent building blocks: (i) leadership and governance; (ii) strategy and investment; (iii) services and applications; (iv) standards and interoperability; (v) infrastructure; (vi) legislation, policy and compliance; and (vii) workforce and capacity building [25]. These building blocks informed the structure of the strategy document as well as the thematic organization of the workshop breakout sessions. Second, the convergence workshop methodology was guided by the ADB Digital Health Convergence Meeting Tool Kit [20], which outlines a six-stage process comprising Request, Coordination, Planning, Preparation, Implementation, and Follow-up. Together, these frameworks enabled a structured and participatory approach to the development of Georgia’s National DHS. Because the WHO–ITU framework structured both the workshop and the deductive arm of the subsequent analysis, the potential for analytic circularity is acknowledged and discussed in Sections 2.5 and 4.6.

### 2.4. Strategy development methodology

Georgia’s DHS development followed a four-phase, multi-step participatory methodology consistent with that described by Patil et al. [24] and adapted for the WHO European Region context (Fig 1). The four-phase methodology described in this section governs the overall strategy-development cycle; the six-stage workshop methodology described in Section 2.4 is nested within Phase 3 and governs the convergence workshop itself.

**Fig 1:**
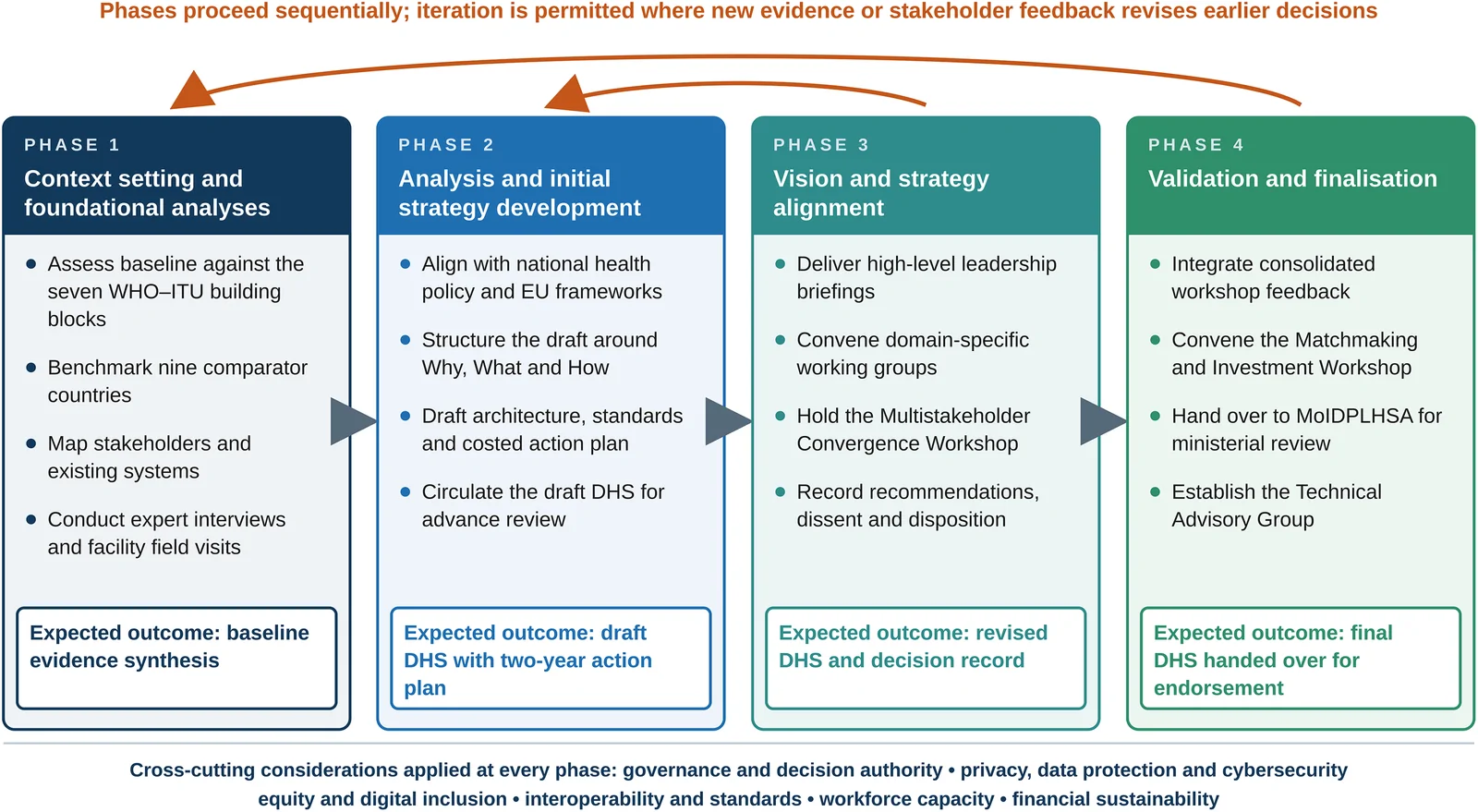
Four-phase, multi-step participatory methodology for developing Georgia’s National Digital Health Strategy. The four phases proceed sequentially, with iteration permitted where new evidence or stakeholder feedback revises earlier decisions: (1) context setting and foundational analyses; (2) analysis and initial strategy development; (3) vision and strategy alignment, incorporating the Multistakeholder Convergence Workshop that is the focus of this paper; and (4) validation and finalisation. Each panel lists the principal steps undertaken and the expected outcome of that phase; cross-cutting considerations applied at every phase are shown beneath. Abbreviations: DGA, Digital Governance Agency; DHS, Digital Health Strategy; EU, European Union; ITA, Information Technology Agency; ITU, International Telecommunication Union; MoIDPLHSA, Ministry of Internally Displaced Persons from the Occupied Territories, Labour, Health and Social Affairs; WHO, World Health Organization. Figure drawn de novo by the authors using original vector shapes and text; it contains no third-party images, icons, or clip-art, and is therefore publishable under the CC BY 4.0 licence. The phase structure follows the participatory blueprint development methodology described by Patil et al. [24], contextualized for Georgia. The figure is supplied as a separate file (Fig1.tif).

**Phase 1—Context setting and foundational analyses.** A baseline assessment was conducted against the seven WHO–ITU building blocks. Global benchmarking identified nine comparator countries, selected against three criteria: (i) regional peers with shared post-Soviet health-system legacies (Armenia, Türkiye); (ii) EU digital health leaders relevant to Georgia’s accession trajectory (Denmark, Estonia, Germany, and the United Kingdom); and (iii) global leaders with published national digital health blueprints (Australia, India, Singapore) [12–19]. Benchmarking followed a structured desk review: each country’s published strategy was reviewed against the seven building blocks using a standardized extraction matrix, distilling design lessons and common pitfalls (for example, weak governance structures, poorly defined action plans, and weak alignment with information and communication technology strategies) that Georgia’s draft sought to avoid. Stakeholder mapping identified key actors across MoIDPLHSA, the ITA, Digital Governance Agency (DGA), Public Service Development Agency (PSDA), National Centre for Disease Control and Public Health (NCDC), National Health Agency (NHA), Georgian Medical Holding (GMH), the State Regulation Agency for Medical and Pharmaceutical Activities (SRAMA), academic institutions, professional associations, private providers, and development partners.

**Phase 2—Analysis and initial strategy development.** A draft DHS was developed, structured around Why (rationale, situational analysis), What (vision, architecture, standards, infrastructure, legislation, governance, capacity), and How (implementation strategy, financial implications, action plan), aligned with the National Health Care Strategy of Georgia 2022–2030 [26] and EU digital health frameworks, including the EHDS Regulation [11]. The draft was informed by semi-structured expert interviews with ministry officials, sub-ministerial agencies, healthcare providers, insurers, academic institutions, and development partners, and by field visits to public and private health facilities; these engagements surfaced current-state constraints and future aspirations and were used to refine successive drafts and build early ownership [27]. Because these consultations were undertaken as programmatic activities rather than research procedures, an exact enumeration of interviews and visits was not maintained as research data; their conduct is documented in the programme record [27]. The draft was additionally informed by a review of grey literature on Georgia’s digital health ecosystem [8–10].

**Phase 3—Vision and strategy alignment.** This phase included high-level briefings, domain-specific working groups (architecture, governance, standards, capacity, implementation), and the Multistakeholder Convergence Workshop that constitutes the focus of this paper.

**Phase 4—Validation and finalization.** Consolidated workshop feedback—collated, coded, and synthesized as described in Section 2.5—was integrated into a revised draft, followed by a Digital Health Matchmaking and Investment Workshop in September 2025 [27] to align prioritized activities with development partner financing, and formal handover to MoIDPLHSA.

### 2.5. Convergence workshop methodology

The Multistakeholder Convergence Workshop was designed using the six-stage ADB framework (Fig 2) [20]. The objectives were to: (i) present the draft DHS; (ii) facilitate in-depth, chapter-wise review through dedicated breakout sessions; (iii) validate key components against national priorities, digital transformation goals, and stakeholder perspectives; (iv) gather structured feedback on implementation priorities, timelines, and resource requirements; (v) identify gaps and challenges across governance, infrastructure, capacity, standards, and interoperability; (vi) validate coordination mechanisms by mapping roles and responsibilities; and (vii) foster consensus on shared goals.

**Fig 2:**
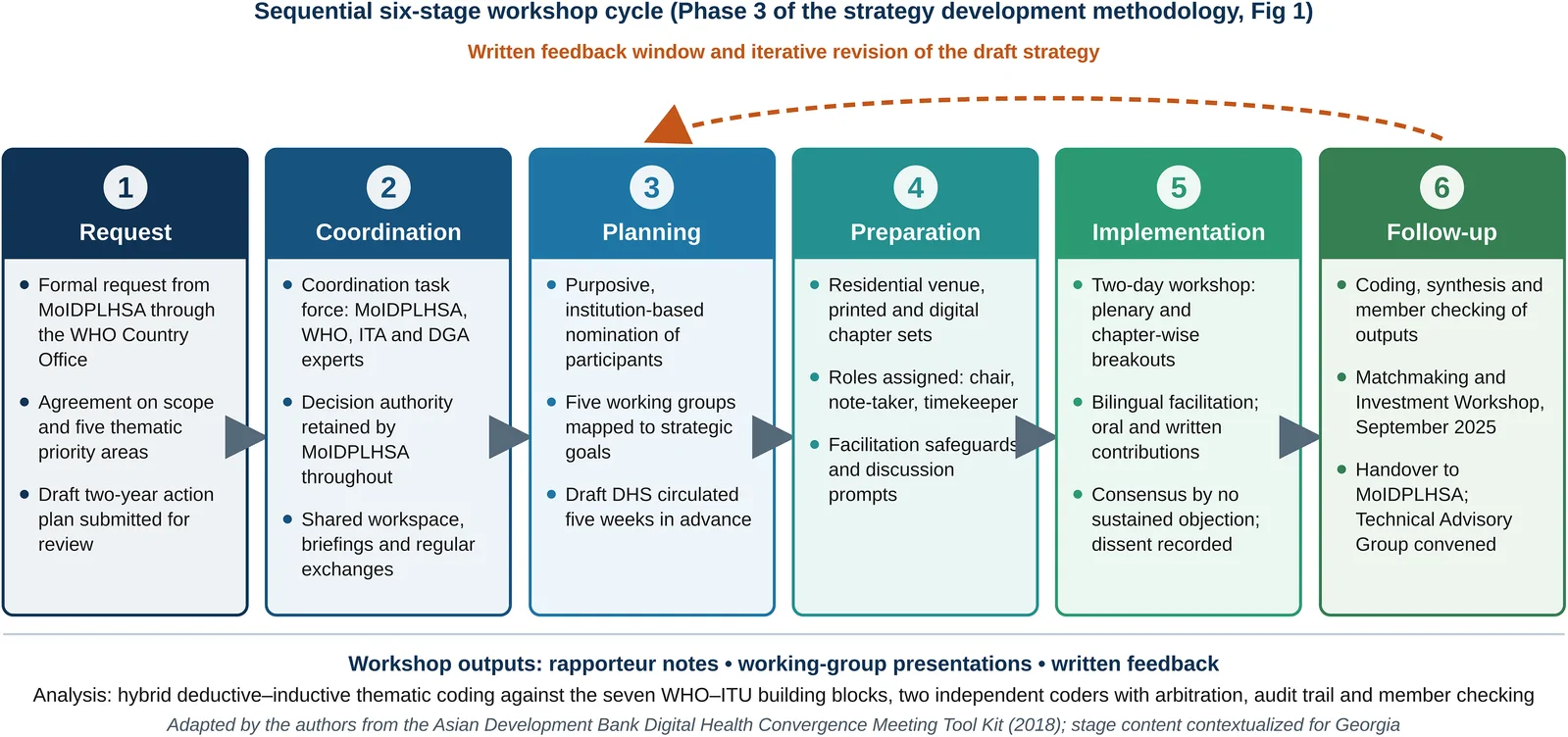
Six-stage convergence workshop methodology as applied in Georgia, adapted from the ADB Digital Health Convergence Meeting Tool Kit (2018) [20]. The six stages—Request, Coordination, Planning, Preparation, Implementation, and Follow-up—constitute the fifth step of Phase 3 of the methodology shown in Fig 1. Each panel summarizes what was undertaken at that stage in Georgia; the dashed arc denotes the written feedback window and iterative revision of the draft strategy following the workshop. The documentation streams generated and the analytic procedure applied to them are shown beneath. Abbreviations: ADB, Asian Development Bank; DGA, Digital Governance Agency; DHS, Digital Health Strategy; ITA, Information Technology Agency; ITU, International Telecommunication Union; MoIDPLHSA, Ministry of Internally Displaced Persons from the Occupied Territories, Labour, Health and Social Affairs; WHO, World Health Organization.

#### 2.5.1. Stage 1—Request.

The workshop was officially requested by MoIDPLHSA in collaboration with the WHO Country Office in Georgia. Five thematic priority areas were defined for review: (i) vision, mission, and guiding principles; (ii) digital health architecture and infrastructure; (iii) governance, legislation, and policy frameworks; (iv) digital health standards; and (v) capacity building and digital literacy. These areas were derived from the chapter architecture of the draft DHS—itself organized around the WHO–ITU building blocks—and consolidated into five groups to keep each group at a deliberative size (8–10 participants). Participants were also asked to review the draft two-year action plan and its financial implications. The draft circulated at this stage carried a strategy horizon to 2027 with a two-year action plan; the extension of the horizon to 2030 was an outcome of the workshop (Section 3.3.5). A concept note was developed outlining the workshop objectives, planned activities, expected outcomes, and proposed stakeholder participation.

#### 2.5.2. Stage 2—Coordination.

Coordination was jointly led by MoIDPLHSA and the WHO Country Office in Georgia, with strategic direction and technical oversight provided by the Strategic Development and Analytics Department of MoIDPLHSA, the WHO Regional Office for Europe (hereafter WHO/Europe), and additional support from WHO–SEARO. A coordination task force comprising MoIDPLHSA representatives, WHO staff, and technical experts from the ITA and DGA was formed. Coordination and communication were facilitated through regular email exchanges, virtual briefing sessions, and a shared digital workspace. Pre-workshop briefing sessions were also conducted to align stakeholder expectations and support chapter-specific preparation and review. Throughout the process, MoIDPLHSA retained final decision-making authority over the content of the DHS: workshop recommendations were advisory; revisions to the strategy were authorized by the Strategic Development and Analytics Department of MoIDPLHSA; and unresolved disagreements were referred to the same department for adjudication or onward legal review.

#### 2.5.3. Stage 3—Planning.

The two-day workshop agenda was developed for 18–19 July 2025 (venue: Sighnaghi; see Stage 4) and brought together 47 participants across six stakeholder categories: (i) MoIDPLHSA and its affiliated agencies; (ii) cross-government bodies (Ministry of Justice, DGA, ITA, PSDA); (iii) the education sector (notably Tbilisi State Medical University); (iv) healthcare providers and professional associations; (v) private-sector providers and health-technology vendors; and (vi) development partners (WHO Country Office, WHO/Europe, WHO–SEARO); the distribution across working groups is reported in Section 3.1 and Table 1. Participants were identified through purposive, institution-based selection: institutions identified through the stakeholder mapping (Section 2.3) were invited by MoIDPLHSA and the WHO Country Office in Georgia to nominate representatives with relevant thematic expertise. Invitation refusals and non-attendance were not systematically recorded. Five thematic working groups were constituted and aligned with the strategic goals of the DHS (Table 1). In addition to their assigned thematic areas, all groups were tasked with reviewing Chapter 3 (Vision, Mission, and Guiding Principles) in recognition of its cross-cutting relevance. The draft DHS document was circulated to participants on 15 June 2025 for review and feedback prior to the workshop.

**Table 1:** Working groups, thematic areas, chapter mappings, and participant numbers.

Group	Thematic area	DHS chapters reviewed	Strategic goal in action plan	Institutions (n)	Participants (n)
1	Digital Health Architecture and Infrastructure	Chapters 3, 4, 7	Goal 1: Strengthening Foundation, Intelligent Systems and Convenient Services	7	9
2	Institutional Framework; Legislation, Policies and Governance	Chapters 3, 5, 8	Goal 2: Enabling Digital Health Policy and Regulatory Framework; Goal 5: Robust Governance	5	10
3	Standards for Digital Health	Chapters 3, 6	Goal 3: Establishing Seamless and Secure Health Data Exchange Standards	7	10
4	Building Capacities for Digital Health	Chapters 3, 9	Goal 6: Empowered People	7	8
5	Implementation Strategy and Financial Implications	Chapters 3, 10	Strategy Action Plan (2026–2030)	7	10
**Total**				**33**	**47**

DHS: Digital Health Strategy. Note: because several institutions were represented in more than one working group, the total of 33 reflects group-level institutional representation rather than unique institutions. Goal 4 (Future-ready IT Framework), which concerns cross-government infrastructure alignment, was reviewed within the remits of Groups 1 and 5.

#### 2.5.4. Stage 4—Preparation.

A residential venue was secured at Hotel Bodbe, Sighnaghi, with equipped meeting rooms and coordinated transportation from Tbilisi; each working group received printed copies of the relevant strategy chapters, and all participants received the full draft DHS in digital form. To facilitate structured engagement and documentation, collaborative digital platforms, standardized templates for presentations and reporting, and chapter-specific discussion prompts were developed to guide deliberation. Within each working group, participants were assigned defined roles—including chair, note-taker, and timekeeper—and were supported by designated facilitators from MoIDPLHSA, the WHO Country Office in Georgia, and WHO–SEARO. Facilitation safeguards were incorporated into the workshop design, including balanced group composition, pre-assigned facilitation prompts, and structured time-management mechanisms to support equitable participation and prevent any single stakeholder or institution from disproportionately influencing discussions; equitable participation was supported by these design features but was not formally measured.

#### 2.5.5. Stage 5—Implementation.

The workshop was conducted over a two-day period. Day 1 commenced with opening remarks from the organizing institutions, followed by a comprehensive presentation of the draft DHS. Participants subsequently transitioned into thematic working groups for two structured sessions of approximately 3.5 hours each. Each session began with a 10–15-minute facilitator-led presentation outlining the session objectives, assigned chapters, and discussion guidelines. Using predefined discussion prompts, participants critically assessed the relevance, clarity, feasibility, and contextual appropriateness of the proposed strategic priorities, implementation actions, and governance frameworks, encouraging cross-sectoral dialogue and practical reflection grounded in Georgia’s health system context.

Inclusive participation and data capture. Inclusive participation was supported through concrete mechanisms rather than assumed: within each group, defined roles distributed responsibility; facilitators used the pre-assigned prompts to invite each represented institution to comment in turn; and written response templates allowed participants to contribute in writing—in Georgian or English—so that quieter participants were not dependent on claiming speaking time. Contributions were captured in written form by designated note-takers drawn from the participants, supported by WHO facilitators, and recorded non-attributably at the level of the working group. No audio recording was made.

Language and facilitation. Although plenary sessions and the formal documentation were conducted in English, breakout deliberations proceeded bilingually where needed: the designated facilitators and note-takers from MoIDPLHSA and the WHO Country Office in Georgia were bilingual Georgian–English speakers and provided ad hoc peer interpretation, and participants could contribute orally or in writing in Georgian, with key points translated into the English-language record. The majority of participants in every group were Georgian nationals representing Georgian institutions, structurally limiting the scope for international-agency voices to dominate deliberations. No professional simultaneous interpretation was provided, and the effectiveness of these measures was not formally assessed; residual language-related bias is discussed in Section 4.6.

Consensus and disagreement management. For the purposes of the workshop, a recommendation was considered adopted by consensus when no sustained objection remained after moderated discussion (a no-objection rule); unanimity was not required, and no voting, ranking, or prioritization scoring was used at this workshop (structured scoring was applied later, at the Matchmaking and Investment Workshop; Section 3.3.5). Facilitators actively invited dissenting views, and minority, contested, or unresolved positions were recorded in the rapporteur notes; these are reported in Section 3.5. Day 2 began with continued thematic group deliberations, followed by plenary presentations from each working group, moderated open discussions, and consensus-building conducted under the same no-objection rule. By the conclusion of the workshop, a consolidated set of stakeholder-informed recommendations and proposed revisions had been generated for integration into the subsequent iteration of the DHS.

#### 2.5.6. Stage 6—Follow-up.

Workshop outputs were systematically documented through structured rapporteur notes, group presentations, and plenary discussion summaries, and subsequently integrated into the revised iteration of the DHS following the vetting and decision-authority arrangements described in Sections 2.4.2 and 3.4. To support the operationalization and financing of identified priorities, a subsequent Digital Health Matchmaking and Investment Workshop was convened in September 2025, bringing together government stakeholders and development partners to align strategic priorities with potential funding and implementation opportunities [27]. In parallel, a Technical Advisory Group was established in the immediate post-workshop period as part of Stage 6 follow-up, institutionalizing the workshop’s recommendation for standing technical oversight; the Group monitors implementation of the workshop-derived priorities, provides ongoing strategic and technical guidance, conducts quarterly review meetings, and supports adaptive management throughout the implementation phase [27].

### 2.6. Data collection and synthesis

Workshop deliberations were collected through structured rapporteur notes, group slide presentations, and written feedback submitted during and after the workshop. Each working group used a standardized reporting template that captured participant roles and contributions, chapter-specific responses to predefined discussion prompts, identified gaps and challenges, key recommendations, and proposed revisions to the action plan. For this analysis, workshop recommendations were thematically mapped against the seven WHO–ITU digital health building blocks using thematic analysis following Braun and Clarke [28]. A hybrid coding design was applied: deductive coding against the seven building blocks—which also structured the workshop and the draft strategy—was complemented by inductive coding to surface themes falling outside the framework (for example, the patient-safety framing of interoperability and contested terminology in the vision statement). Two members of the author team (NG, MG) coded all source material independently and in parallel; a third author (KA) acted as arbiter where the two coders’ initial classifications differed, and remaining discrepancies—principally concerning whether a recommendation pertained to one or two building blocks—were resolved through discussion to consensus. A coding matrix recording each recommendation, its source group, source document type, building-block assignment, cross-cutting status, and disposition was maintained in spreadsheet form throughout, constituting the audit trail for the synthesis; this matrix is published as S1 Data. No qualitative data analysis software was used. Findings were triangulated across the three independent documentation streams (rapporteur notes, group presentations, and written feedback). Formal inter-rater reliability statistics were not computed, because coding units were recommendation-level statements resolved by discussion; this is acknowledged in Section 4.6.

Cross-cutting priorities were defined as recommendations emerging independently in two or more working groups; this threshold is the minimal criterion for multi-group emergence, avoids overstating convergence given only five groups, and maintains consistency with the prior multi-country application of the methodology [24], preserving comparability. Group-specific priorities were those raised in a single group, reflecting distinct thematic concerns. After completion of the synthesis, the synthesized findings were shared with the working-group chairs and the designated WHO focal points for member-check validation, to verify that the synthesis accurately reflected each group’s deliberations; their corrections were incorporated. Because the coding framework was applied retrospectively to programme documentation, and because the WHO–ITU framework structured both the workshop and the deductive analysis, framework-consistent convergence is expected by design; this analytic circularity is acknowledged as a limitation in Section 4.6.

### 2.7. Reflexivity

The author team includes staff and consultants of the WHO Country Office in Georgia, WHO/Europe, WHO–SEARO, and MoIDPLHSA who designed, facilitated, and coordinated the process evaluated in this paper. This dual implementer–evaluator role carries a recognized risk of confirmation bias. Mitigation measures adopted for this study were: independent parallel coding by two authors with third-party arbitration (Section 2.5); member-check validation of the synthesis by working-group chairs and WHO focal points; deliberate documentation and reporting of disagreements, rejected proposals, and unresolved issues (Section 3.5); a before–after change mapping that anchors evaluative statements in verifiable differences between the pre-workshop draft and the revised strategy (Table 6); systematic moderation of evaluative language throughout the manuscript; and open publication of the full coding matrix (S1 Data), enabling independent scrutiny of the synthesis. No independent external evaluation of the workshop was conducted, and this remains a limitation (Section 4.6).

## 3. Results

### 3.1. Workshop characteristics and stakeholder participation

The Multistakeholder Convergence Workshop convened 47 participants across five thematic working groups (Table 1). Working groups ranged in size from 8 to 10 participants, and each group represented 5–7 distinct institutions. Stakeholder representation spanned six categories: government and policy-making (MoIDPLHSA Strategic Development and Analytics Department, Department of Health Care Policy, regulation unit); public health and IT agencies (ITA, NCDC, NHA, DGA, PSDA, SRAMA, GMH); academic institutions (Tbilisi State Medical University); professional associations (Georgian Family Medicine Professionals’ Association, Healthcare Association, Institute of Healthcare Transformation); private-sector providers and health-technology vendors (Geo Hospital, Jerarsi Clinic, Medsoft, Radium); and development partners. Each working group was supported by a designated WHO facilitator and assigned roles for chair, note-taker, and timekeeper. Because several institutions were represented in more than one working group, the column total of 33 in Table 1 reflects group-level institutional representation rather than the number of unique institutions. The professional-role distribution of individual participants was not systematically recorded.

### 3.2. Validation of vision, mission, and guiding principles

All five working groups reviewed the cross-cutting Chapter 3. The proposed vision—*“A trusted, secure, connected and accessible digital health ecosystem that empowers all people of Georgia to live healthier, safer lives”*—and the corresponding mission—*“To develop a person-centric and inclusive digital health ecosystem that offers convenient, secure and quality services to all health system participants, and is built on an interoperable architecture that fosters cost-effectiveness, collaboration and innovation”*—were endorsed by all five working groups, with no group proposing rejection or fundamental redrafting; the refinements proposed are itemized below.

Specific refinements proposed during the workshop and subsequently incorporated into the revised draft included: (i) replacing “all Georgians” with “all citizens of Georgia” for inclusivity (Group 3)—the final DHS ultimately adopted the broader formulation “all people of Georgia”, encompassing citizens and non-citizen residents alike; (ii) adding “quality” as an explicit attribute in the mission statement (Group 4); (iii) clarifying or replacing “affordable”, which was perceived as primarily financial in connotation (Groups 3, 4, 5); and (iv) adding “secure” as a distinct attribute alongside “trusted” (Group 5). Further refinements included: (v) renaming Goal 3 from “Seamless Health Data Exchange” to “Seamless and Secure Health Data Exchange” with a corresponding security objective (Group 3); (vi) amending Goal 6 to strengthen individuals’ rights of access to and control over their personal health data (characterized by participants as “ownership”; the final DHS frames this in terms of data-subject rights of access and control rather than legal ownership of data) (Group 3); and (vii) revising Goal 4 terminology from “to accelerate” to “to align” for foundational digital public infrastructure such as the Instant Payment System and Digital ID, recognizing that MoIDPLHSA can ensure alignment but not accelerate the development of cross-government infrastructure (Group 2). Group 5 additionally recommended explicit reference to Georgia’s signing of the Council of Europe Framework Convention on Artificial Intelligence and stronger emphasis on data sovereignty, research, and quality assurance; this recommendation resulted in explicit reference to the Convention in the guiding principles and the inclusion of artificial intelligence (AI) governance within the scope of the proposed Digital Health Act (Section 3.3.2).

### 3.3. Synthesis of working-group recommendations

Working-group recommendations were thematically mapped against the seven WHO–ITU eHealth Strategy building blocks (Table 2). Across all groups, several convergent priorities emerged, including: governance strengthening, FHIR-based interoperability, foundational standards adoption (LOINC, ICD-11), data-protection legislation, and integrated workforce capacity-building. Group-specific recommendations are summarized in Sections 3.3.1–3.3.5.

**Table 2:** Synthesis of Multistakeholder Convergence Workshop recommendations organized by WHO–ITU eHealth Strategy Building Blocks. Entries are recommendations as advanced by the working groups; their subsequent disposition in the revised DHS is summarized in Table 6 and Section 3.4. Phased entries are labelled immediate, medium-term, or long-term to distinguish near-term specifications from long-term aspirations.

Building block	Workshop recommendations (Georgia)
1. Leadership and governance	• Adopt a federated data governance model, replacing the existing decentralized arrangement (Group 1). • Establish a Steering Committee chaired by at least the First Deputy Minister and a Technical Working Group, anchored in an amended ministerial decree, within 2–3 months of strategy launch (Group 2). • Set up a dedicated Digital Health Unit within MoIDPLHSA in the medium term, hosted under the Strategic Development and Analytics Department or the Department of Health and Care Policy (Group 2). • Defer establishing a stand-alone digital health agency; reassess after the two-year implementation period (Group 2). • Define an organogram for the Digital Health Unit covering strategy, standards, applications, legislation, and capacity (Group 2).
2. Strategy and investment	• Extend the strategy horizon from 2027 to 2030, with a detailed two-year action plan (2026–2027) (Group 5). • Address financial sustainability through diverse sources (state budget, donor, public–private partnerships) and incentive mechanisms (tax relief, innovation grants, co-financing), advanced as options for further costing and feasibility analysis (Group 5). • Establish a formal mid-2026 review checkpoint to recalibrate based on implementation evidence (Group 5). • Embed adaptive management mechanisms across the action plan (Group 5). • Avoid duplication of foundational digital public infrastructure already operational at national level (e.g., Instant Payment System, Digital ID) (Group 5).
3. Services and applications	• Establish a national Health Information Exchange (HIE) as a foundational service for accurate, secure data exchange (Group 1). • Reclassify common building block ‘Analysis’ from ‘good-to-have’ to ‘essential’ (Group 1). • Conduct a comprehensive assessment and mapping of existing digital health applications, in order to address misalignments with the proposed domain building blocks and to identify functional overlaps and gaps (Group 1).
4. Standards and interoperability	• Use the existing national personal identification number rather than introducing a separate Health ID, supported by privacy safeguards including a privacy impact assessment, pseudonymization for secondary use, and controls against cross-domain linkage risks (Group 1). • Immediate (0–12 months): establish a National Standards Management Entity and multi-stakeholder Standards Advisory Committee; adopt foundational standards including FHIR-based data exchange, LOINC, electronic signature, and digital ID (Group 3). • Medium term (1–3 years): develop a National Interoperability Action Plan; launch a national standards catalogue and developer portal with technical specifications, APIs, implementation guides, and adoption toolkits (Group 3). • Long term (3 + years): institutionalize a standards lifecycle governance model; transition to an opt-out consent model (modelled on Estonia)—recorded as a legal and data-governance policy measure requiring legislative change rather than a technical standard; advance toward implementation of FHIR and WHO-FIC classifications together with complementary clinical terminologies (e.g., SNOMED CT) supported by mandatory certification (Group 3). • Approve medical consumables classifiers through legislation as part of foundational digital public infrastructure, to standardize procurement and supply-chain data (Group 4).
5. Infrastructure	• Address current and projected data storage and processing needs alongside cybersecurity, data backup, and business continuity planning, fully integrated into budgeting (Group 1). • Participants from the ITA assessed current data center capacity as only marginally sufficient and anticipated it would be increasingly stressed by analytics rollout (Group 1). • Strengthen network connectivity and human resource capacity in high-mountain and rural municipalities (Group 1). • Conduct cost–benefit analyses for integration with the Georgian Government Gateway (3G) and the National Bank of Georgia’s Instant Payment System before integration commitments (Group 1). • Strengthen digital infrastructure and software support at the PHC level to ensure equitable access (Group 4).
6. Legislation, policy, compliance	• Draft a stand-alone Digital Health Act covering data lifecycle management, primary and secondary use of health data, interoperability, data protection, cybersecurity, and AI governance (Group 2). • Restructure legal recommendations into clearly distinguished categories: amendments to existing acts and new provisions under a Digital Health Act (Group 2). • Shift legal monitoring from the National Digital Health Governance Council to the Agency for Regulation of Medical and Pharmaceutical Activities (Group 2). • Assign oversight of secondary use of health data to Data Protection Officers, in consultation with the Georgian Statistical Office—advanced as a proposal requiring legal feasibility assessment (Group 2). • Remove outdated or unverifiable legal references (e.g., 1997 Law on Health Insurance) (Group 2). • Resolve regulatory inconsistencies including parallel paper-and-EMR documentation requirements under existing orders (Group 4). • Prioritize implementation of electronic signatures (Group 4).
7. Workforce and capacity building	• Establish a national committee to lead design, development, and oversight of digital health competency frameworks (Group 4). • Conduct baseline research and evaluation of current national digital health capacities (Group 4). • Develop role-specific practice guidelines distinguishing baseline and advanced competencies; integrate digital health training into existing Continuing Professional Development programs (Group 4). • Introduce postgraduate (master’s and doctoral) programs in digital health, public health, healthcare management, IT, and health informatics, with digital health competencies embedded in their curricula (Group 4). • Establish formal curriculum review and update mechanisms within health-related academic programs (Group 4). • Adopt a population-wide approach to digital health literacy as the initial phase, complemented by targeted support for digitally excluded and vulnerable groups (Group 4). • Incentivize PHC and rural service providers to lead digital health awareness initiatives (Group 4).

AI: artificial intelligence; API: application programming interface; CPD: Continuing Professional Development; DHS: Digital Health Strategy; EMR: electronic medical record; FHIR: Fast Healthcare Interoperability Resources; HIE: Health Information Exchange; ICD-11: International Classification of Diseases, 11th Revision; ICF: International Classification of Functioning, Disability and Health; ICHI: International Classification of Health Interventions; IT: information technology; ITA: Information Technology Agency; LOINC: Logical Observation Identifiers Names and Codes; PHC: primary health care; SNOMED CT: Systematized Nomenclature of Medicine – Clinical Terms; WHO-FIC: World Health Organization Family of International Classifications.

#### 3.3.1. Group 1: Digital health architecture and infrastructure.

Group 1, comprising representatives of ITA, NCDC, NHA, PSDA, the Agency for Regulation of Medical and Pharmaceutical Activities, and MoIDPLHSA’s Strategic Development and Analytics Department, endorsed without recorded objection a federated data governance model as the best fit for Georgia, citing the predominance of privately owned facilities (~86% of hospital beds), the heterogeneity of vendor systems across those facilities, and existing decentralized data-management practices [8]. Participants distinguished federated data governance—distributed accountability for data stewardship across autonomous public and private entities operating under common national rules—from the federated technical architecture that would support it; the recommendation encompassed both. The Health Information Exchange (HIE) was endorsed as the architectural mechanism enabling secure exchange among decentralized systems under that governance model, and as a core foundational component for seamless, accurate, and secure health data exchange. Crucially, the group rejected the development of a separate Health ID, arguing that all citizens are already registered using the existing national personal identification number; introducing a parallel Health ID was deemed redundant and inefficient. The group noted that reuse of the national identification number as the health identifier would require privacy safeguards, including a privacy impact assessment, pseudonymization for secondary use, and controls against cross-domain linkage risks.

The group recommended a comprehensive assessment and mapping of existing digital health applications to address misalignments with proposed domain building blocks and to identify functional overlaps and gaps. On infrastructure resilience, participants from the ITA assessed current data center capacity as only marginally sufficient to meet demand and anticipated it would be increasingly stressed by analytics rollout; budget and resource allocation should explicitly include cybersecurity, data backup, and business continuity planning. The group also highlighted limited human resource capacity and inadequate network speed in high-mountain municipalities as critical constraints, requiring infrastructure investment and workforce strengthening in parallel. Regarding integration with foundational digital public infrastructure—the Georgian Government Gateway (3G) and the National Bank of Georgia’s Instant Payment System—the group called for feasibility studies and structured cost–benefit analyses prior to integration commitments. The Instant Payment System, in particular, should be evaluated for adherence to open standards, open specifications, and modular open APIs.

#### 3.3.2. Group 2: Institutional Framework and Legislation, Policies and Regulations.

Group 2, comprising MoIDPLHSA Strategic Development and Analytics Department, Department of Health Care Policy, NCDC, and NHA, agreed with the proposed short-term governance model—a Steering Committee at decision-making level chaired by at least the First Deputy Minister and a Technical Working Group at implementation level. However, the group did not support an immediate dedicated digital health agency, citing risks of overlapping mandates with ITA and DGA, the absence of national consensus on standards, and participants’ assessment of a shortage of digital health professionals—illustrated, among other indicators, by the absence of master’s programs in health informatics in Georgia and consistent with the ADB assessment [9]. Reassigning ministry staff to a new agency was perceived as likely to weaken existing departments; further, the ITA’s mandate extends beyond health to broader social, labor, and IDP issues.

Instead, the group proposed establishing a Digital Health Unit within MoIDPLHSA in the medium term, hosted under either the Strategic Development and Analytics Department or the Department of Health and Care Policy, with responsibilities spanning strategy coordination and implementation, standards and digital architecture, digital health applications, legislation, and capacity building. Re-evaluation of a stand-alone digital health agency was deferred to after the two-year implementation period.

On legislation, the group recommended structural rationalization of the chapter into clearly distinguished categories: amendments to existing acts, and new provisions under a proposed stand-alone Digital Health Act. The Digital Health Act would cover data lifecycle management, primary and secondary use of health data, interoperability, data protection, cybersecurity, and AI governance. Participants noted that the proposed Act would consolidate and complement—rather than duplicate—existing instruments, including the Law of Georgia on Personal Data Protection and general cybersecurity legislation, by addressing health-sector-specific matters not covered by horizontal law. Specific recommendations included shifting legal monitoring from the National Digital Health Governance Council to the Agency for Regulation of Medical and Pharmaceutical Activities; assigning oversight of secondary use of health data to Data Protection Officers in consultation with the Georgian Statistical Office—advanced as a proposal requiring legal feasibility assessment (Section 3.5); and removing outdated or unverifiable legal references (e.g., the 1997 Law on Health Insurance and unclear ministerial decrees). The group additionally recommended licensing pathways for digital health tools through recognition of trusted-jurisdiction usage—that is, recognition of digital health tools already authorized in designated trusted jurisdictions (for example, the EU/European Economic Area)—and acceptance of international certifications, likewise subject to legal feasibility review, and integration of findings from the most recent State Office Audit on EHR compliance. All legal recommendations were advanced as workshop proposals subject to formal legal feasibility review during drafting.

#### 3.3.3. Group 3: Standards for digital health.

Group 3, comprising representatives of DGA, ITA, NCDC, MoIDPLHSA, and private vendors (Medsoft, Radium), confirmed that Georgia’s current standards landscape is characterized by continued reliance on ICD-10—notwithstanding the global transition toward ICD-11—fragmented terminology, and absence of FHIR-based or digital lab reporting standards. Participants described, in anonymized and non-attributable form, real-world instances in which fragmented records disrupted care—for example, treatment interruptions where prior chemotherapy records could not be retrieved—resulting in treatment delays and repeat diagnostics. Stakeholders concluded that interoperability is not only an efficiency concern but a patient-safety imperative. In discussing the standards stack, participants distinguished the WHO-FIC reference classifications (ICD-11; the International Classification of Functioning, Disability and Health, ICF; and the International Classification of Health Interventions, ICHI) from SNOMED CT, a clinical terminology; LOINC, a coding system for laboratory and other observations; and FHIR, an interoperability standard and exchange specification.

The group strongly endorsed a phased roadmap. In the immediate term (0–12 months), a National Standards Management Entity and multi-stakeholder Standards Advisory Committee should be established, alongside adoption of foundational standards including digital ID, electronic signature, FHIR-based data exchange, and LOINC for laboratory data. In the medium term (1–3 years), a National Interoperability Action Plan should be developed and a national standards catalogue and developer portal launched—providing centralized, public access to technical specifications, APIs, implementation guides, and adoption toolkits. In the long term (3 + years), Georgia should institutionalize a standards lifecycle governance model and advance toward implementation of FHIR and WHO-FIC together with complementary terminologies such as SNOMED CT, supported by structured governance, implementation guidance, and quality assurance mechanisms. Participants recognized that transition to ICD-11 is a multi-year undertaking requiring terminology mapping, coder training, information-system migration, and a period of dual coding. A proposed transition to an opt-out consent model (as practiced in Estonia [14]) was recorded as a legal and data-governance policy measure requiring legislative change—rather than a technical standard—and was referred to the legislative workstream (Section 3.3.2). The group also raised, but did not resolve, whether national SNOMED CT licensing should be sought independently or in alignment with the EU4Digital regional framework (Section 3.5).

#### 3.3.4. Group 4: Building capacities for digital health.

Group 4, comprising MoIDPLHSA’s Department of Health Care Policy, Tbilisi State Medical University, the Georgian Family Medicine Professionals’ Association, the Healthcare Association, Jerarsi Clinic, and the Agency for Regulation of Medical and Pharmaceutical Activities, recommended establishing a national committee to lead the design and oversight of digital health competency frameworks, alongside baseline research and evaluation to assess current digital health capacities. Role-specific practice guidelines should distinguish baseline from advanced competencies, with priority given to integrating digital health training modules into existing Continuing Professional Development (CPD) programs.

The group recommended introducing postgraduate programs (master’s and doctoral) in public health, healthcare management, IT, and health informatics, with digital health competencies embedded in their curricula, to prepare the future workforce. Inclusion of digital health competencies in undergraduate medical education was endorsed as a longer-term objective requiring curriculum reform, regulatory alignment, and stakeholder coordination. Formal curriculum review and update mechanisms within health-related academic programs were recommended to ensure alignment with evolving digital health needs. The group emphasized the necessity of practical, task-specific software skills—such as navigating EMRs and telemedicine platforms—rather than generic digital competency, particularly for clinical staff, and flagged the parallel paper-and-EMR documentation requirement under Order 108 as an active disincentive to digital adoption.

For population-level digital health literacy, the group endorsed a broad-based public approach as the initial phase, rather than the population-segment-specific model in the draft, complemented by targeted support for digitally excluded and vulnerable groups (for example, older adults and rural populations), with targeted awareness initiatives to be revisited after a population-wide baseline is established. Critically, the group recommended incentivizing primary healthcare and rural service providers to lead local digital health awareness efforts—including potentially through carefully designed key performance indicators (KPIs) for family physicians, an approach participants noted would require safeguards against unintended effects such as tokenistic reporting or workload distortion (Section 3.5). The group also flagged that full implementation of training programs within a two-year timeframe was unrealistic, requiring phased planning; this concern contributed directly to the recommendation to extend the strategy horizon to 2030 (Section 3.3.5).

#### 3.3.5. Group 5: Implementation strategy and financial implications.

Group 5, comprising NCDC, MoIDPLHSA, NHA, DGA, the WHO Country Office, the Institute of Healthcare Transformation, and a private hospital financial director, recommended extending the strategy horizon from 2027 to 2030, with a detailed two-year action plan (2026–2027) outlining short- and medium-term milestones. The action plan should include clearly defined monitoring and evaluation (M&E) ownership, data flow pathways, reporting schedules, feedback mechanisms, component-specific budget estimates, and risk and mitigation strategies. The group flagged that several baseline impact indicators recorded values of 0% because the corresponding interventions had not yet commenced; such baselines were considered uninformative for trend assessment, and the group recommended refining them with measurable interim milestones.

Financial sustainability should be addressed through diverse sources—state budget, donor funding, and public–private partnerships (PPPs)—supported by incentive mechanisms such as tax relief, innovation grants, and co-financing schemes to attract private-sector engagement; these mechanisms were advanced as options for further costing and feasibility analysis rather than as validated solutions. The Georgian Innovation and Technology Agency (GITA) and venture-funding mechanisms were highlighted as additional avenues for innovation support. A formal mid-2026 implementation review checkpoint should be established to allow recalibration based on emerging evidence—participants acknowledged that the checkpoint’s timing would depend on the strategy’s formal approval date—and adaptive management should be embedded throughout the action plan. The group also recommended detailing PPP modalities through concrete use cases and aligning the legal and risk-sharing framework with EU practice. The 38 priority activities were subsequently prioritized through a structured exercise combining multi-criteria decision analysis with the nominal group technique, conducted at the September 2025 Matchmaking and Investment Workshop [27].

### 3.4. Outputs and timeline

Following the workshop, recommendations were vetted and integrated into the revised DHS under the decision-authority arrangements described in Section 2.4.2: recommendations were advisory, and a ministerial stakeholder group—comprising representatives of the strategy and finance divisions—conducted additional consultations with lead agencies before revisions were authorized; not all workshop outputs were integrated verbatim, and the disposition of each recommendation is recorded in S1 Data. The revised strategy was reframed around six strategic goals and 38 priority activities (Table 3). The strategy was presented at a Digital Health Matchmaking and Investment Workshop convened in September 2025 to align prioritized activities with development partner financing [27] and was formally handed over to MoIDPLHSA in late 2025; at the time of writing, the strategy was undergoing final governmental review and formal endorsement was pending. The full development cycle—from MoIDPLHSA’s initial request to handover—spanned approximately six to eight months.

**Table 3:** Strategic goals and indicative priority activities of Georgia’s National Digital Health Strategy 2026–2030.

Goal	Strategic goal	Indicative priority activities
1	Strengthening Foundation, Intelligent Systems and Convenient Services of Digital Health Architecture	Design, develop, and implement core and common building blocks of the Digital Health Ecosystem (DHE) Technology Stack; assess and align existing digital health applications with the proposed DHE Technology Stack; establish a national Health Information Exchange; transition to a federated data governance model; conduct comprehensive assessment and mapping of existing applications.
2	Enabling Digital Health Policy and Regulatory Framework	Establish a Digital Health Act covering data lifecycle, interoperability, data protection, cybersecurity, secondary data use, and AI governance; operationalize the Act through delegated legislation and enforcement tools; remove outdated legal references; clarify roles between regulatory bodies.
3	Establishing Seamless and Secure Health Data Exchange Standards	Drive governance and develop a unified national strategy for adoption, implementation and maintenance of data and digital standards; advance toward implementation of FHIR, WHO-FIC together with complementary terminologies; develop a national standards catalogue and developer portal; institutionalize a standards lifecycle governance model.
4	Future-ready IT Framework	Drive equitable and sustainable access to digital devices for the MoIDPLHSA and health facility workforce; align with the national Digital ID; align with the national Instant Payment System; integrate the Georgian Government Gateway (3G) with the DHE Technology Stack.
5	Robust Governance	Institutionalize a Steering Committee and Technical Working Group through an amended ministerial decree; establish a dedicated Digital Health Unit within MoIDPLHSA in the medium term; develop a clear organogram and terms of reference; reassess the case for a stand-alone digital health agency after the two-year implementation period.
6	Empowered People	Establish standardized digital health competency frameworks for the health workforce and population groups; conduct national baseline assessment of digital health competencies; design role-specific training programs for the existing workforce (basic and advanced tiers) integrated into CPD; introduce postgraduate digital health programs; implement population-wide digital health literacy initiatives, with PHC providers incentivized to lead local awareness efforts.

AI: artificial intelligence; CPD: Continuing Professional Development; DHE: Digital Health Ecosystem; FHIR: Fast Healthcare Interoperability Resources; IT: information technology; MoIDPLHSA: Ministry of Internally Displaced Persons from the Occupied Territories, Labour, Health and Social Affairs; PHC: primary health care; WHO-FIC: World Health Organization Family of International Classifications.

To distinguish changes attributable to the workshop from pre-existing draft content: the six strategic goals and the chapter architecture of the strategy predated the workshop and were carried into the revised DHS. The workshop validated this overall structure while producing specific, documented modifications; the principal workshop-attributable changes between the pre-workshop draft and the post-workshop revised DHS are summarized in Table 6. The draft circulated before the workshop contained approximately 45 candidate activities, which were consolidated into the 38 priority activities of the revised strategy during post-workshop refinement.

### 3.5. Areas of disagreement and unresolved issues

Consistent with the consensus definition in Section 2.4.5, most recommendations were adopted without sustained objection; the following elements generated disagreement or remained unresolved and were documented in the workshop record. First, Group 3 raised, but did not resolve, whether national SNOMED CT licensing should be pursued independently or in alignment with the EU4Digital regional framework; the question was referred to the standards workstream. Second, the Georgian translation and connotation of “affordable” in the vision statement was contested across Groups 3, 4, and 5, being perceived as narrowly financial; the term was flagged for revision rather than agreed. Third, Group 2 explicitly diverged from the draft’s proposal for a dedicated digital health agency, rejecting immediate establishment in favour of a Digital Health Unit within MoIDPLHSA—the clearest instance of the workshop overturning a draft provision. Fourth, the proposal to assign oversight of secondary use of health data to Data Protection Officers was not settled on its merits and was referred for legal feasibility review. Fifth, participants cautioned that KPI-linked incentives for family physicians could produce unintended effects and required careful design. Sixth, the realism of the original two-year implementation timeline was directly challenged by Group 4, driving the horizon extension adopted through Group 5. We note that the modest volume of recorded disagreement beyond these items may partly reflect consensus-oriented facilitation and the non-attributable documentation approach; this possibility is acknowledged as a limitation in Section 4.6.

## 4. Discussion

### 4.1. Principal findings

This study suggests that a structured convergence-based methodology, originally developed in an Asian context, can be adapted to support national digital health strategy development in an upper-middle-income WHO European Region country pursuing EU alignment. Across five thematic working groups, the workshop produced technically specific, contextually grounded recommendations that were vetted and integrated into a revised DHS. Several convergent priorities mirror those reported across settings and can be considered broadly universal: governance strengthening, FHIR-based interoperability, infrastructure optimization through reuse of existing digital public infrastructure (DPI) rather than duplicative stand-alone investments, explicit data-protection legislation, and workforce capacity-building integrated into pre-service and in-service training pathways, aligning with prior applications of the convergence methodology in Maldives, Nepal, Timor-Leste, Sri Lanka, and Kenya [21–24].

By contrast, other recommendations were distinctly Georgia-specific: a federated data governance model suited to a predominantly private healthcare delivery landscape; rejection of a separate Health ID in favor of the existing national personal identification number; institutional alignment via a Digital Health Unit within MoIDPLHSA rather than a stand-alone agency; explicit alignment with the EHDS Regulation [11] and EU4Digital frameworks; and adaptive implementation extended to 2030 with a formal mid-2026 review checkpoint.

### 4.2. Comparison with existing literature

Our findings align with an emerging evidence base identifying governance, interoperability, workforce, and legal frameworks as recurrent enablers of national digital health scale-up across low- and middle-income countries and—as Georgia’s upper-middle-income experience suggests—in health systems undergoing structural transition more broadly [24,29,30]. The recurrent recommendation to adopt FHIR-based architectures and internationally recognized classifications and terminologies (LOINC; WHO-FIC, including ICD-11, ICF, ICHI) echoes documented practice in interoperability specifications and clinical terminology development [31,32] and is consistent with the direction of the EHDS Regulation [11], although the Regulation does not itself mandate the specific terminologies discussed here. Emphasis on pragmatic reuse of existing DPI aligns with the broader World Bank and United Nations positioning on digital public infrastructure for development [33,34]: reuse of shared infrastructure reduces capital duplication and long-term maintenance burden, which is what obviates the case for dedicated digital health data centers. This also underscores the importance of maintaining the WHO-FIC as the globally governed foundation for health data standardization, reporting, and international comparability.

Georgia’s experience extends prior convergence workshop findings in several notable ways. First, the outputs were technically detailed across all thematic groups—encompassing a federated data governance model, use of the existing national ID as primary health identifier, a phased standards roadmap with explicit timelines, and structured cost–benefit analyses for foundational DPI integration—although, in the absence of a formal comparative analysis, we do not claim that this level of detail exceeds that of earlier applications of the ADB toolkit [20–22]. Second, the workshop placed strong emphasis on institutional and legal anchoring, advancing concrete proposals including amendments to ministerial decrees and the drafting of a stand-alone Digital Health Act. This resonates with Kenya’s experience, where the convergence process preceded and informed the development of the Digital Health Act (2023) and its implementing regulations [35,36], suggesting that legal frameworks are central to effective digital health implementation, supporting continuity and enforceability across political cycles.

Third, the integration of financial sustainability and infrastructure resilience was distinctive. In addition to PPP models and donor coordination, recommendations included explicit measures for cybersecurity, data backup, and business continuity planning, alongside an adaptive management plan with a mid-2026 review checkpoint. Fourth, the capacity-building emphasis was broad and inclusive: while many countries focus primarily on competencies within the existing workforce, Georgia’s recommendations encompassed integration of digital health into postgraduate education, CPD programs, and population-level digital literacy through PHC-led awareness initiatives.

Finally, hospital-level digital maturity assessment emerged as a complementary avenue not used in this cycle. Maturity instruments such as the HIMSS Electronic Medical Record Adoption Model (EMRAM) and Infrastructure Adoption Model (INFRAM) [37] provide standardized, facility-level staging of digitalization and are widely used in comparative assessments across the WHO European Region. Whereas the WHO–ITU building blocks structure strategy at the system level, EMRAM/INFRAM-style baselines would be particularly valuable in Georgia, where the central implementation challenge is integrating a predominantly private hospital sector with heterogeneous levels of digitalization. Incorporating such maturity assessment into future situational analyses—and into monitoring of the interoperability roadmap—would strengthen both benchmarking and implementation tracking.

### 4.3. Adaptation to the WHO European Region context

Several aspects of Georgia’s application of the convergence methodology reflect adaptation to the WHO European Region context. First, the strategy is deliberately framed against the EHDS Regulation [11] and EU4Digital programme expectations, shaping recommendations on cross-border data exchange, standards harmonization, and consent management. In this context it is useful to distinguish four levels that jointly shaped the strategy’s framing: binding EU law (including the EHDS Regulation, directly applicable to EU Member States); Georgia’s voluntary pre-accession alignment agenda; EU4Digital programme support to Eastern Partnership countries; and WHO/Europe regional policy as articulated in the Regional Digital Health Action Plan 2023–2030 [7].

Second, comparator benchmarking incorporated regional peers (Armenia, Türkiye), EU digital health leaders (Denmark, Estonia, Germany, the United Kingdom), and global leaders (Australia, India, Singapore) [12–19], consistent with the Regional Digital Health Action Plan’s emphasis on peer learning across countries at different levels of digital maturity. Third, the strategy explicitly recognizes Georgia’s signing of the Council of Europe Framework Convention on Artificial Intelligence; the Convention’s specific effect was the addition of an explicit reference in the guiding principles and the incorporation of AI governance within the scope of the proposed Digital Health Act. Together, these features suggest that the convergence methodology may be portable beyond its original Asian context with appropriate adaptation, consistent with prior observations in settings beyond Asia and Africa [24]; we note, however, that this remains a single European case, and further applications are needed before portability can be asserted with confidence. Overall, the integration of both EU policy frameworks and WHO regional priorities demonstrates the relevance of aligning national strategies with multilevel governance structures to ensure coherence, scalability, and sustainability in digital health transformation.

### 4.4. Toward an expanded stakeholder model

While the workshop achieved broad representation across core institutional actors, structured post-workshop reflection by the author team—rather than the workshop deliberations themselves—identified groups whose voices were under-represented: frontline primary care providers, patient advocacy groups, civil society organizations (which were not among the six represented stakeholder categories), and small health-technology innovators. Table 4 is accordingly an author-derived, post hoc recommendation and proposes an expanded stakeholder model for future convergence workshops in similar settings, distinguishing core actors from currently under-represented actors whose structured inclusion would enhance technical rigor, equity orientation, and implementability. The absence of these voices carries concrete implementation risks for 2026–2030: provisions that touch clinical documentation workflows—resolution of the parallel paper–EMR requirement, EMR adoption at PHC level, and KPI-linked incentives for family physicians—are precisely those most likely to encounter point-of-care resistance if designed without frontline input, while population-facing components (digital health literacy, patient-facing services) risk misalignment with citizens’ actual preferences and digital access. The Technical Advisory Group’s quarterly review cycles, subsequent public consultations, and PHC-focused rollout activities are the concrete vehicles through which these missing perspectives should be structurally captured going forward, using the model proposed in Table 4.

**Table 4:** Expanded stakeholder model for future multistakeholder convergence workshops on digital health strategies. Author-derived recommendation based on structured post-workshop reflection (Section 4.4).

Core actors (consistently represented in Georgia)	Recommended expansion	Rationale for inclusion
Senior MoH representatives (MoIDPLHSA, Strategic Development and Analytics Department, Health Care Policy Department)	National digital governance and IT authorities (DGA, PSDA)	Secures alignment with whole-of-government digital public infrastructure and reduces risk of duplicative investments.
Public health and regulatory agencies (NCDC, NHA, GMH, SRAMA)	National standards-development and accreditation bodies; the Personal Data Protection Service of Georgia (data-protection supervisory authority)	Ensures technical and legal enforceability of standards and certification pathways.
WHO Country Office; WHO/Europe; WHO–SEARO	EU Delegation, bilateral and multilateral development partners (EBRD, ADB, World Bank, USAID, GIZ), other UN agencies (UNICEF, UNFPA, UNOPS)	Aligns national priorities with international financing and technical assistance, supporting matchmaking.
National IT agencies, enterprise architects (ITA, DGA)	Frontline health workforce: PHC physicians, nurses, midwives, community-level providers	Grounds draft content in service-delivery realities and surfaces workflow constraints.
Professional associations (e.g., family medicine, healthcare association)	Private-sector providers, insurers (the NHA as public purchaser, alongside private insurers), and health-technology start-ups	Reflects heterogeneity of service delivery and financing in Georgia’s predominantly private system.
Medical universities (e.g., TSMU)	Patient advocacy groups and civil society organizations	Strengthens equity, user-centered design, and public trust in digital health deployments.
	**Public-health student bodies**	**Builds the future digital health workforce pipeline and brings emerging-professional perspectives.**

ADB: Asian Development Bank; DGA: Digital Governance Agency; EBRD: European Bank for Reconstruction and Development; GIZ: Deutsche Gesellschaft für Internationale Zusammenarbeit; GMH: Georgian Medical Holding; IT: information technology; MoH: Ministry of Health; MoIDPLHSA: Ministry of Internally Displaced Persons from the Occupied Territories, Labour, Health and Social Affairs; NCDC: National Centre for Disease Control and Public Health; NHA: National Health Agency; PHC: primary health care; PSDA: Public Service Development Agency; SEARO: South-East Asia Regional Office; SRAMA: State Regulation Agency for Medical and Pharmaceutical Activities; TSMU: Tbilisi State Medical University; UN: United Nations; USAID: United States Agency for International Development; WHO: World Health Organization.

### 4.5. Lessons learnt and recommendations for replication

Beyond the expanded stakeholder model, structured reflection on Georgia’s experience yielded a set of operational lessons and recommendations for replication in other WHO European Region Member States and comparable upper-middle-income settings (Table 5). These lessons were generated through a structured post-workshop debrief of the coordination team and author reflection during manuscript preparation; the numeric parameters cited (briefing durations, feedback windows, sequencing intervals) are indicative starting points drawn from this single experience rather than validated standards. Several recommendations—particularly on pre-workshop preparation, structured feedback elicitation, and local-language facilitation—are consistent with parallel applications of the methodology in non-European contexts [24], suggesting transferable lessons.

**Table 5:** Lessons learnt and recommendations for replicating the convergence methodology in other settings. Parameters are indicative rather than validated standards.

Dimension	Lessons learnt (Georgia)	Recommendation for replication
Pre-workshop preparation	Although the draft DHS was circulated five weeks before the workshop, the depth of advance review varied across participants and groups.	Circulate pre-read materials at least four weeks in advance with chapter-specific reading guides and concise briefing notes for each WHO–ITU Building Block; conduct approximately 60–90-minute pre-workshop briefings per group.
Feedback elicitation	Structured prompts were prepared, but the discussion sometimes diverged from assigned chapters under time pressure.	Use structured elicitation templates keyed to the assigned Building Block; include explicit prompts on relevance, feasibility, and priority; train designated rapporteurs in advance.
Language and facilitation	English-only facilitation in some sessions limited engagement of non-English-speaking participants; translation of key terms (e.g., ‘affordable’) was contested in Georgian, notwithstanding the bilingual facilitation arrangements described in Section 2.4.5.	Provide local-language facilitation and/or professional simultaneous interpretation for breakout sessions; ensure bilingual rapporteurs; pre-translate key strategic concepts.
Stakeholder composition	Frontline PHC providers, patient advocacy groups, and small health-tech innovators were underrepresented.	Adopt the expanded stakeholder model (Table 4); use hybrid (in-person and virtual) formats to widen participation without sacrificing deliberative depth.
Technical specificity	Discussions on architecture, interoperability, and standards benefited from deeper technical engagement than a single workshop could accommodate.	Convene supplementary technical working groups on architecture, standards, and data protection before and after the main workshop, feeding consolidated inputs into plenary.
Contextual adaptation	Georgia’s regulatory context (EU alignment, EHDS) required substantial adaptation of comparator benchmarking and legal recommendations from the original ADB toolkit.	Tailor the agenda, expert presenters, and benchmarking comparators to each country’s regional regulatory context; avoid prescriptive cross-country templates.
Implementation horizon	The original two-year action plan was deemed unrealistic for several initiatives (e.g., curriculum reform, postgraduate programs, workforce-wide training).	Match the implementation horizon to the realistic delivery timeline of structural reforms—reforms such as curriculum change and postgraduate-programme establishment typically require multiple years—with phased two-year action plans within.
Post-workshop continuity	Momentum risked dissipating between the workshop, matchmaking event, and finalization, as inferred from the intervals between these milestones.	Establish a time-bound written feedback window (indicatively 10–14 days); formalize working groups as standing technical advisory bodies; convene a matchmaking workshop within approximately 2–3 months.

ADB: Asian Development Bank; DHS: Digital Health Strategy; EHDS: European Health Data Space; EU: European Union; PHC: primary health care; WHO: World Health Organization.

### 4.6. Strengths and limitations

This study has several strengths. It documents, in operational detail, the application of a convergence methodology to a country setting outside its original Asian context. The synthesis was triangulated procedurally—across three independent documentation streams and between two independent coders with third-party arbitration—and validated through member checking; the institutional diversity of the author team (MoIDPLHSA, the WHO Country Office in Georgia, WHO/Europe, WHO–SEARO, and an external technical consortium) supports, though does not by itself constitute, methodological reflexivity. The use of an internationally recognized conceptual framework (WHO–ITU Building Blocks) enables comparability with country experiences elsewhere, and the open publication of the coding matrix (S1 Data) permits independent scrutiny of the synthesis (Table 6).

**Table 6:** Principal changes between the pre-workshop draft and the post-workshop revised Digital Health Strategy attributable to workshop deliberations.

Pre-workshop draft provision	Workshop-attributable change	Disposition in revised DHS
Strategy horizon to 2027 with two-year action plan	Horizon extended to 2030, retaining a detailed two-year action plan (2026–2027) and mid-2026 review checkpoint (Group 5)	Incorporated
Decentralized data governance arrangement	Transition to a federated data governance model, with the HIE as its architectural mechanism (Group 1)	Incorporated
Separate Health ID under consideration	Use of the existing national personal identification number, with privacy safeguards; separate Health ID rejected (Group 1)	Incorporated
Goal 3 titled “Seamless Health Data Exchange”	Renamed “Seamless and Secure Health Data Exchange” with a corresponding security objective (Group 3)	Incorporated
Goal 6 without explicit data-rights language	Strengthened individuals’ rights of access to and control over personal health data (framed as data-subject rights) (Group 3)	Incorporated
Goal 4 phrased “to accelerate” foundational digital public infrastructure	Rephrased “to align”, reflecting MoIDPLHSA’s actual mandate (Group 2)	Incorporated
Dedicated digital health agency proposed	Agency deferred; dedicated Digital Health Unit within MoIDPLHSA established in the medium term instead (Group 2)	Incorporated
Legal provisions distributed across the chapter	Restructured into amendments to existing acts vs. new provisions under a stand-alone Digital Health Act, including AI governance (Group 2; Group 5 for AI reference)	Incorporated
Common building block “Analysis” classified good-to-have	Reclassified as essential (Group 1)	Incorporated
Population-segment-specific digital literacy model	Population-wide approach as initial phase, with targeted support for digitally excluded groups (Group 4)	Incorporated
Approximately 45 candidate activities in the action plan	Consolidated to 38 priority activities during post-workshop refinement, subsequently prioritized via MCDA–NGT at the Matchmaking and Investment Workshop [27]	Incorporated

DHS: Digital Health Strategy; HIE: Health Information Exchange; MCDA: multi-criteria decision analysis; MoIDPLHSA: Ministry of Internally Displaced Persons from the Occupied Territories, Labour, Health and Social Affairs; NGT: nominal group technique. The complete recommendation-level disposition record is provided in S1 Data.

Several limitations warrant acknowledgement. First, the workshop was constrained by a tight two-day timeframe, which limited the depth of deliberation in certain areas, particularly implementation prioritization and financing strategies; the subsequent Digital Health Matchmaking and Investment Workshop was designed to address this gap [27]. Second, participant selection was subject to bias: purposive, institution-based nomination privileges established institutional actors, and representation from civil society, frontline primary care providers, and patient advocacy groups was limited or absent; end-user perspectives—patients’, citizens’, and frontline clinicians’ priorities and preferences regarding their health data—were not directly captured, creating a risk of top-down implementation disconnected from actual usage. Future iterations should adopt the expanded stakeholder model proposed in Table 4. Third, English-language facilitation may have limited the depth of engagement for some Georgian-speaking participants, notwithstanding the bilingual arrangements described in Section 2.4.5, whose effectiveness was not formally assessed.

Fourth, the deliberative setting itself carries biases that the design could mitigate but not eliminate: hierarchy and power dynamics within groups containing senior officials, social desirability pressures in a ministry-convened forum, and the possibility of facilitator influence given that facilitation was provided by WHO and MoIDPLHSA staff. Fifth, the analysis is subject to framework-confirmation bias: the WHO–ITU building blocks structured the draft, the workshop, and the deductive arm of the coding, so framework-consistent (normative) recommendations are expected by design, and the study cannot fully distinguish genuinely emergent participatory priorities from reproduction of pre-existing international standards; the inductive coding arm and the documentation of draft-divergent outcomes (Table 6; Section 3.5) partially, but only partially, offset this. Sixth, the qualitative synthesis was applied retrospectively to programme documentation rather than under a prospective protocol; although dual independent coding, arbitration, an audit trail, and member checking were used, no formal inter-rater reliability statistics were computed. Seventh, no objective, quantitative measure of consensus was applied at the workshop: the no-objection rule documents the absence of sustained dissent, not its strength or distribution. Eighth, several authors designed and implemented the process they evaluate here; the mitigation measures are described in Section 2.6, but no independent external evaluation was conducted. Finally, this case study describes a single cycle of strategy development; longitudinal evaluation of endorsement-to-implementation trajectories will be needed to test whether convergence outputs translate into sustained implementation—implementation effectiveness remains untested. Translating technically rich outputs into sustained implementation will depend on continued political will, cross-sector coordination, and predictable financing—factors that fall outside the workshop’s direct scope.

### 4.7. Implications for practice and policy

For WHO European Region Member States and comparable settings advancing digital health strategies, the findings suggest that structured convergence workshops could be considered for institutionalization as a discrete phase within national strategy development cycles, contingent on further comparative evaluation across settings; a single case cannot ground a universal recommendation. Ministries of Health and WHO Country Offices may find it useful to combine the six-stage ADB workshop framework with the four-phase blueprint development methodology, while tailoring stakeholder composition, language, comparator benchmarking, and substantive content to country-specific governance, regulatory, and digital maturity contexts.

Separately, at regional and global levels, partners should consider updating the 2018 ADB Digital Health Convergence Meeting Tool Kit [20] to reflect contemporary developments in digital public infrastructure, AI governance, the European Health Data Space [11], and contemporary data-protection regulation.

## 5. Conclusion

The development of Georgia’s first National DHS through a structured Multistakeholder Convergence Workshop represents an important milestone toward a more integrated, inclusive, and future-ready health system. Anchored in international best practice, contextualized through systematic comparator benchmarking, and refined through broad—though not fully representative—multi-stakeholder dialogue, the process supported both technical development and national ownership of the strategy. The workshop contributed to a revised strategy comprising six strategic goals and 38 priority activities and shifted the strategy horizon from 2027 to 2030 with a phased action plan and adaptive management framework. Georgia’s experience suggests that the convergence methodology, originally developed for Asian Member States, can be adapted to upper-middle-income WHO European Region countries pursuing EU alignment, while implementation effectiveness remains untested. Refinements in stakeholder breadth, language facilitation, post-workshop continuity, and longitudinal evaluation would further enhance generalizability. These findings contribute to the wider evidence base on national-level digital health strategy development and offer a potentially transferable approach for similar settings advancing digital health agendas.

## Supporting information

S1 Data Anonymized aggregated thematic coding matrix of Multistakeholder Convergence Workshop recommendations. Recommendation-level records with source working group, WHO–ITU building-block mapping, cross-cutting classification, disposition in the revised DHS, and documented areas of disagreement.
(XLSX)
